# Supplementary material for: Complexome profiling of the Chlamydomonas psb28 mutant reveals TEF5 as an early PSII assembly factor
Source: Plant Cell. 2025 Mar 17;37(6):koaf055. doi: 10.1093/plcell/koaf055 (PMC12164590; doi:10.1093/plcell/koaf055)
Supplement: koaf055_Supplementary_Data [file koaf055_supplementary_data.zip › psb28_interactive.zip!/plots/intensity/qualityPlots/peptides/cob_Cre-2.g801484.t1.p1.html]

### Cre-2.g801484.t1.p1

Trivial name: cob  
SeqCoverage [median]: 2.90  
Mass: 42.30 kDa  
  
 

| Sequence | MissedCleavage | Mass [Da] | wtIntensity | muIntensity | Score |
| --- | --- | --- | --- | --- | --- |
| GMYYGSGAQPR | 0 | 1185.52 | 4379.92 | 2115.65 | 113.71 |
|  |  |  | 4379.9 | 2115.6 |  |
